# Impact of Inactivated Influenza Vaccination on Health‐Related Quality of Life Among Japanese Healthcare Workers

**DOI:** 10.1002/iid3.70266

**Published:** 2025-09-19

**Authors:** Taito Kitano, Sayaka Yoshida

**Affiliations:** ^1^ Department of Pediatrics Nara Prefecture General Medical Center Nara Japan; ^2^ Division of Infection Control Nara Prefecture General Medical Center Nara Japan

**Keywords:** health personnel, influenza, quality of life, quality‐adjusted life years, safety, vaccination

## Abstract

**Background:**

Influenza vaccinations are recommended for healthcare workers (HCWs). Quantification of the personal risks of vaccination compared to vaccine benefits can help guide more accurate benefit‐risk assessment and cost‐effectiveness analysis of inactivated influenza vaccination among HCWs. The study objective was to evaluate the quality‐adjusted life day (QALD) loss due to adverse events following immunization (AEFI) among HCWs.

**Methods:**

This study used a questionnaire survey with the EuroQol‐5 dimension‐5 level (EQ‐5D) for HCWs to evaluate the impact of reactogenicity on QALD loss following inactivated seasonal influenza vaccination. Participants were asked to answer a questionnaire survey regarding their health status once daily from the day before vaccination until 7 days after vaccination (a total of 9 times). QALD loss was calculated as the cumulative difference between the mean EQ‐5D scores following vaccination (Days 0–7) and the mean EQ‐5D score before vaccination (Day −1).

**Results:**

During the study period, 213 participants completed the surveys for 5 days or more, and 122 participants completed the surveys for all days. The mean QALD losses among the participants who completed the surveys for five or more days and those who completed the surveys on all days were 0.040 and 0.054, respectively. The mean QALD losses among those by grade (the maximal grade of any AEFIs) were 0.021 (grade 0), −0.018 (grade 1), 0.089 (grade 2), and 0.299 (grade 3), respectively.

**Conclusion:**

We measured the magnitude of QALD loss in HCWs following inactivated influenza vaccination. These results support a more accurate health technology assessment of seasonal influenza vaccination in this population.

## 1 Introduction

Influenza primarily causes respiratory infections and is associated with a variety of consequences, including cardiovascular events and exacerbation of underlying medical conditions [1]. The disease burden of influenza is enormous, particularly for patients with underlying medical conditions [2, 3]. Healthcare workers (HCWs) play a critical role in providing healthcare services for those with influenza, as well as in potentially transmitting influenza from patient to patient [4].

Therefore, influenza vaccination is recommended for HCWs [5]. The main purpose of vaccinating HCWs against influenza is to protect patients and HCWs from influenza, as well as to prevent disruption of healthcare services due to the absenteeism of HCWs [6]. In addition, vaccine hesitancy against influenza has also been reported among HCWs [7, 8, 9]. This illustrates that the quantification of personal risks of vaccination, compared with vaccine benefits using a single health outcome scale, can help guide rationale decision making on inactivated influenza vaccination for HCWs.

Quality‐adjusted life years (QALY) or quality‐adjusted life days (QALD), calculated from the duration and utility weight (or utility value) of a health state, are often used to quantify health outcomes [10]. QALY is also fundamental for benefit‐risk assessments and cost‐effectiveness analyses of vaccinations [11, 12]. Although benefit‐risk assessment and cost‐effectiveness of vaccination for HCWs are vital not only from a societal perspective but also from the perspectives of individual HCW's and healthcare facilities, quantitative data regarding the risks of influenza vaccination among HCWs, such as QALY loss or QALD loss, are scarce.

Reactogenicity outcomes, including local symptoms such as pain and redness at the injection site, as well as systemic symptoms such as fever, myalgia, and headache, usually present and subside within 7 days of vaccination [13]. Some studies have shown that reactogenicity outcomes account for the majority of QALY losses following vaccination [14]. Adverse events following immunization (AEFIs), including reactogenicity, are often graded [15, 16]. For example, grade 3 AEFI was associated with significant QALD loss following recombinant zoster vaccination in the elderly [17]. The objective of this study was to evaluate QALD loss following influenza vaccination among HCWs.

## 2 Materials and Methods

### 2.1 Study Design and Settings

This study used a questionnaire survey using the EuroQol‐5 dimension‐5 level (EQ‐5D‐5L) for HCWs in Nara Prefecture General Medical Center, Japan, between 21 October and November 24, 2024, to evaluate the impact of reactogenicity following inactivated influenza vaccination on QALD loss. Eligible participants were HCWs who received a 0.5‐mL dose of inactivated non‐recombinant quadrivalent seasonal influenza vaccine during the study period without any other vaccination 7 days before or after the influenza vaccination. The study center administered an annual influenza vaccination to HCWs who wished to be vaccinated. All eligible participants in the study center were offered to participate in the electronic questionnaire study by providing a study information sheet. On the information sheet, a study introduction paragraph was included, which stated that (1) the study was completely voluntary, (2) participants' anonymity was maintained, (3) anonymized results were to be published, and (4) those answering the survey provided consent to participate in the study. The survey was conducted electronically using SurveyMonkey. All participants completed the survey using their smartphones or digital devices. The study protocol, with a waiver of written informed consent, was reviewed and approved by the Institutional Review Board of Nara Prefecture General Medical Center, Japan.

Participants were asked to answer a questionnaire survey regarding their health status once daily from the day before vaccination until 7 days after vaccination (a total of 9 times). The questionnaire asked about participants' age group, sex, chronic comorbidities, daily local and systemic symptoms by grade, and daily health‐related quality of life using the EQ‐5D‐5L instrument. The AEFIs were graded (grade 0 none, grade 1 mild, grade 2 moderate, grade 3 severe, and grade 4 life‐threatening or urgent interventions indicated) based on the guidance of the US Food and Drug Administration [18, 19]. Participants were asked to evaluate their health status at the end of the day. Vaccination was administered during the day, and their answers on Day 0 of vaccination reflected their health status after vaccination.

The EQ‐5D‐5L is a validated instrument used to measure the health state utility value (HSUV) score in adults on a given day. It consists of five dimensions (mobility, self‐care, usual activities, pain and discomfort, and anxiety and depression), and participants were asked to select their degree of problem in each dimension by five levels [20]. The answers for the five dimensions were converted into a single summary of the HSUV score (1 = perfect health and 0 = death). Japanese scoring data were used to convert the participants' answers in the EQ‐5D‐5L to HSUV scores with a time trade‐off method [21]. Daily HSUV measurements using the EQ‐5D‐5L instrument were conducted in multiple previous studies to evaluate the impact of vaccination [16, 17, 19, 22, 23]. The instrument also asked participants about their visual analog scale (VAS), with 0 and 100 being the worst and best health states they could imagine, respectively. The use of EQ‐5D‐5L Japanese version for this study was approved by EuroQol. The minimal important difference of the EQ‐5D score for the Japanese population was 0.061 [24].

### 2.2 Statistical Analysis

The mean EQ‐5D and EQ‐VAS scores among participants who answered the daily questionnaire for 5 days or longer (given that most reactogenicity symptoms resolved within 4 days) [25] and for all days (from Day −1 to Day +7 of vaccination) were calculated. QALD loss was calculated as the area under the curve of difference between the mean EQ‐5D scores following vaccination (Days 0–7) and the mean EQ‐5D score before vaccination (Day −1) using trapezoidal rule [26]. QALY was calculated as QALD/365.25. To assess the difference in EQ‐5D score in each day (Day 0 to 7) following vaccination, compared with those before vaccination (Day −1), Wilcoxon signed‐rank test was conducted. The Bonferroni correction was applied to interpret the statistical significance of the eight comparisons (cut‐off *p*‐value = 0.05/8).

Subgroup analysis was conducted using the maximal grade of AEFIs. A sensitivity analysis was conducted by including all study participants, irrespective of the number of answer days in the daily questionnaire.

To evaluate the impact of covariates on QALY loss, a generalized linear model was performed with identity link function. The covariates included participant's age (< 40 years vs. 40 years or older), sex, presence of any chronic comorbidity, and history of influenza vaccination (receipt of influenza vaccination in the last season.

Stata release 18 (StataCorp) and Microsoft Excel 2019 (Redmond) were used for the statistical analyses.

## 3 Results

During the study period, 1149 HCWs received inactivated influenza vaccinations, and 280 participants (24.4%) agreed to answer the questionnaire and provided their answers in the first survey (pre‐vaccination; Day −1), of which 80.7% were female, and 79.6% did not have any chronic comorbidities. Of these, 213 participants completed the surveys for 5 days or more, and 122 participants completed the surveys for all days (from Day −1 to Day +7 of vaccination). The participants' backgrounds are listed in Table 1.

**TABLE 1:** Participants backgrounds.

	All participants	Participants completed answered for > = 4 days	Participants completed answered for all days
*N*	280	213	122
Age (years)			
18–29	65 (23.2%)	36 (16.9%)	22 (18.0%)
30–39	71 (25.4%)	53 (24.9%)	29 (23.8%)
40–49	57 (20.4%)	47 (22.1%)	26 (21.3%)
50–59	64 (22.9%)	57 (26.8%)	34 (27.9%)
> = 60	23 (8.2%)	20 (9.4%)	11 (9.0%)
Gender (Women)	226 (80.7%)	172 (80.8%)	98 (80.3%)
Chronic comorbidity			
None	223 (79.6%)	167 (78.4%)	95 (77.9%)
Respiratory	2 (0.7%)	1 (0.5%)	1 (0.8%)
Cardiovascular, including hypertension	10 (3.6%)	8 (3.8%)	5 (4.1%)
Kidney	2 (0.7%)	2 (0.9%)	1 (0.8%)
Liver	2 (0.7%)	2 (0.9%)	1 (0.8%)
Diabetes	5 (1.8%)	4 (1.9%)	1 (0.8%)
Hematologic	2 (0.7%)	1 (0.5%)	1 (0.8%)
Immunosuppressing, including active treatment for malignancy	2 (0.7%)	2 (0.9%)	2 (1.6%)
On immunosuppressant, including steroid	2 (0.7%)	1 (0.5%)	1 (0.8%)
Neurologic or muscular	0 (0.0%)	0 (0.0%)	0 (0.0%)
Psychiatric	5 (1.8%)	4 (1.9%)	1 (0.8%)
Others	29 (10.4%)	23 (10.8%)	15 (12.3%)
No answer	4 (1.4%)	4 (1.9%)	2 (1.6%)
Last dose of influenza vaccine			
1 year ago	268 (95.7%)	204 (95.8%)	116 (95.1%)
2 years ago	3 (1.1%)	2 (0.9%)	2 (1.6%)
3 years ago	4 (1.4%)	3 (1.4%)	2 (1.6%)
> = 4 years ago	5 (1.8%)	4 (1.9%)	2 (1.6%)
Never been vaccinated	0 (0.0%)	0 (0.0%)	0 (0.0%)

**FIGURE 1:**
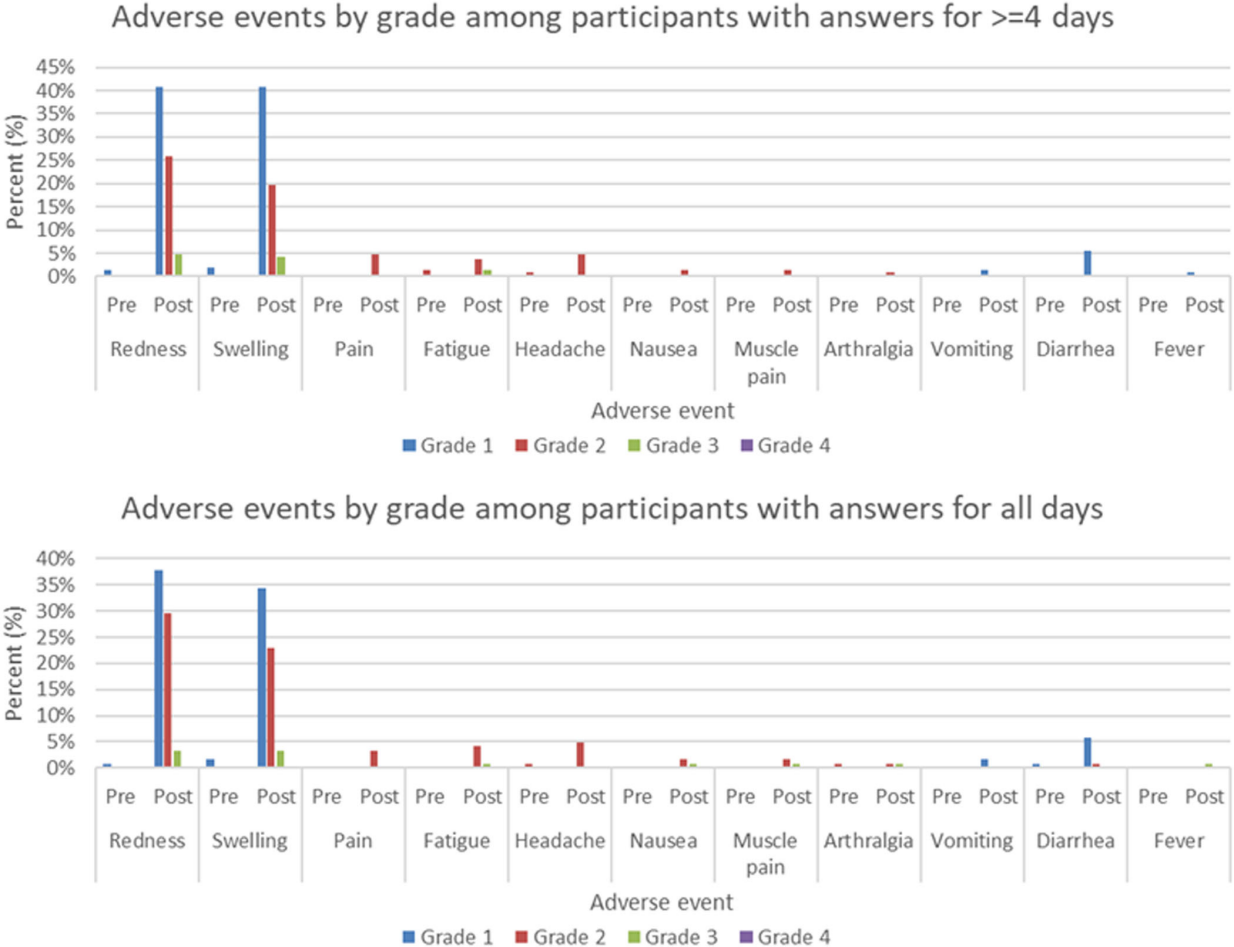
Adverse events by grade. Postvaccination, pre; pre‐vaccination. Grade of postvaccination was the maximal grade in 0–7 days after vaccination.

Among those who completed the surveys for 5 or more days, the maximal grades of any AEFIs were grade 0 (23.5%), grade 1 (39.9%), grade 2 (30.0%), grade 3 (6.1%), and grade 4 (0.5%). The only one participant with grade 4 AEFI developed a fever, which resolved by Day 2 following vaccination. Among those who completed the surveys on all days, the maximum grade of any AEFIs was 0 (27.0%), grade 1 (35.2%), grade 2 (32.8%), and grade 3 (4.9%) (Figure 1).

**FIGURE 2:**
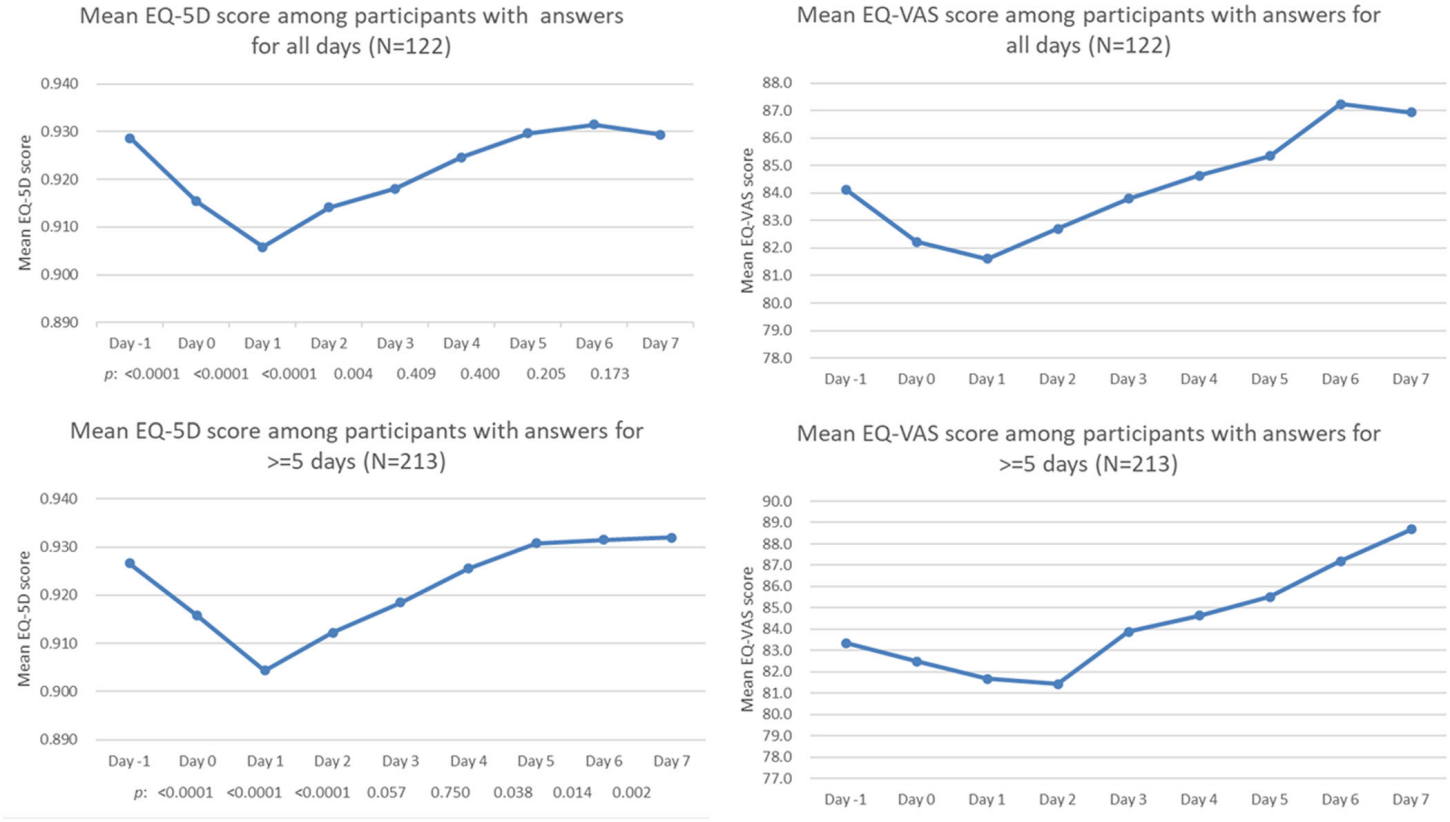
Mean EQ‐5D and EQ‐VAS scores. Mean scores were calculated as the mean in the study population in each day. EQ‐5D, EuroQol 5‐dimension; EQ‐VAS, EuroQol visual analog scale; *p*, *p*‐value.

The mean EQ‐5D and EQ‐VAS scores on the day before and after vaccination are presented in Figure 2. Among participants who completed surveys for ≥ 5 days, EQ‐5D score after vaccination was significantly lower from Day 0 to Day 2 (*p* < 0.001) and greater on Day 7 (*p* = 0.002), compared with that before vaccination (Figure 2). Among the participants who completed surveys on all days, compared with the EQ‐5D scores before vaccination, those after vaccination were significantly lower on Days 0–3 (*p* < 0.001), whereas those on Days 4–7 were not statistically different.

The mean QALD loss among participants who completed the surveys for 5 or more days was 0.040 (0.00011 QALY loss), and that among participants who completed the surveys for all days was 0.054 (0.00015) (Table 2). Subgroup analysis showed that the mean QALD losses among those by grade (the maximal grade of any AEFIs) were 0.019 (grade 0), −0.016 (grade 1), 0.079 (grade 2), and 0.297 (grade 3), respectively. In the sensitivity analysis, the mean QALD loss among all the participants, irrespective of the number of answer days, was 0.044 (Table 3).

**TABLE 2:** Mean quality‐adjusted life day (QALD) or quality‐adjusted life year (QALY) loss.

	Mean QALD loss	Mean QALY loss
Participants with answers for > = 5 days	0.040 [0.006–0.074]	0.00011 [0.00002–0.00020]
Participants with answers for all days	0.054 [0.011–0.098]	0.00015 [0.00003–0.00027]
Subgroup analysis (participants with answers for > = 5 days)		
Participants with grade 0 AEFI after vaccination	0.019 [−0.002–0.039]	0.00005 [0.00000–0.00011]
Participants with grade 1 AEFI after vaccination	−0.016 [−0.052–0.020]	−0.00004 [−0.00014–0.00006]
Participants with grade 2 AEFI after vaccination	0.079 [0.024–0.135]	0.00022 [0.00007–0.00037]
Participants with grade 3 AEFI after vaccination	0.297 [−0.098–0.692]	0.00081 [−0.00027–0.00189]
Sensitivity analysis		
All participants irrespective of the number of answer days	0.044 [0.020–0.068]	0.00012 [0.00005–0.00019]

*Note:* The 95% confidence interval of each mean value was calculated by point estimate +/− 1.96 standard deviation. To calculate standard error, a missing value in a given day was imputed by replacing missing data with the mean of the non‐missing data on the corresponding day of vaccination.

Abbreviations: QALD; quality‐adjusted life day, QALY; quality‐adjusted life year

**TABLE 3:** Generalized linear model for quality‐adjusted life day loss following inactivated influenza vaccination.

	Coefficient [95% confidence interval]	*p*‐Value
Age (< 40 years)	0.05 [−0.05, 0.15]	0.299
Sex (Female)	0.05 [−0.07, 0.16]	0.411
Presence of any chronic comorbidity	0.01 [−0.10, 0.12]	0.856
History of influenza vaccination in last season	−0.30 [−0.52, −0.09]	0.005

AIC = 0.113, BIC = −554.7, *R* ^2^ = 0.09.

*R* ^2^ was calculated by running a linear regression analysis with the same covariates.

The generalized linear model showed that participant's age group (*p* = 0.299 in < 40 years vs. 40 years or older), sex (*p* = 0.411 in females vs. males), and presence of any chronic comorbidity (*p* = 0.856 in those with chronic comorbidity vs. those without) were not significantly associated with QALD loss. The history of influenza vaccination in the last year was associated with fewer QALD loss (coefficient −0.30 [−0.52, −0.09] and *p* = 0.005).

## 4 Discussion

This study evaluated the impact of reactogenicity on HSUV scores among Japanese HCWs following inactivated influenza vaccination using the EQ‐5D instrument. While a significant reduction in the EQ‐5D score was observed in the early days (e.g., Day 0 to 3) following vaccination, the EQ‐5D score in later days (e.g., Day 4 or later) recovered to that in the pre‐vaccination level. This was consistent with the fact that reactogenicity symptoms resolved within a few days of vaccination.

The data regarding QALD loss following inactivated influenza vaccination measured in our study support more accurate evaluations of health technology assessments. For example, cost‐effectiveness assessments of influenza vaccination for HCWs and the general adult population have been conducted in some studies [27, 28, 29, 30]. However, none of these studies considered the impact of AEFI as QALY loss, even though QALY is a critical component of cost‐effectiveness analysis [31]. National and international guidelines encourage the incorporation of the benefits and risks of vaccines into health technology assessments [32, 33]. The results of our study would facilitate the consideration of both benefits and risks of inactivated influenza vaccination for HCWs or the general adult population in future assessments.

Although evidence regarding HSUV change resulting from influenza vaccination is scarce, a previous study showed that the EQ‐5D score was reduced by 0.05 in 2 days postvaccination compared with pre‐vaccination among the elderly population [34]. However, that study measured the HSUV score only once after vaccination, which prevented the evaluation of QALY or QALD loss following influenza vaccination over time. Another study with a design similar to ours showed that a change in EQ‐5D score following simultaneous vaccination with a messenger RNA (mRNA) COVID‐19 vaccine and an inactivated influenza vaccine was significantly different from that following an mRNA COVID‐19 vaccine only [16]. However, that study could not evaluate QALD loss due to influenza vaccination alone. Another study evaluated QALD loss following administration of the adjuvanted monovalent influenza A (H5N1) vaccine, which is not routinely used to prevent seasonal influenza infection [35]. To the best of our knowledge, this is the first study to determine the magnitude of QALD loss following vaccination with inactivated seasonal influenza. The statistical association of a fewer QALD loss with the history of influenza vaccination in the last year needs to be interpreted with caution because of the low value of model fit indicator (*R* ^2^ = 0.09).

Our results by AEFI grade showed that those with severe reactogenicity symptoms (i.e., grade 2 or 3) had larger QALD losses than those with milder symptoms (i.e., grade 0 or 1), demonstrating the face validity of the EQ‐5D instrument in this context. In our study, only 6.1% and 0.5% of participants who completed surveys for ≥ 5 days had grade 3 or 4 AEFI, respectively. This suggests good tolerability of the inactivated influenza vaccine, which is consistent with the results of previous relevant studies [36]. Some vaccines showed a relatively high proportion of severe reactogenic symptoms. For example, grade 3 AEFIs have been reported in 9.5% and 15.6% of elderly vaccinees after the first and second doses of a recombinant zoster vaccine, respectively [17, 19]. They estimated that the QALY loss following the first and second doses of the recombinant zoster vaccine was 0.000064 and 0.00012, respectively [17, 19]. Although the proportion of participants with severe AEFI was lower in our study than in studies of recombinant zoster vaccine in the elderly, the magnitude of QALY loss following vaccination was not smaller in our study. This may be attributed to multiple factors, including differences in the study populations (i.e., HCWs vs. the elderly) and vaccine type (i.e., influenza vs. zoster and non‐recombinant vs. recombinant). For influenza vaccine, a high‐dose recombinant vaccine is often administered, especially for the elderly [37]. Future research includes evaluations of the impact of the recombinant influenza vaccine on the magnitude of QALY change.

Our study had some limitations. First, the response rate and completion rate from Day −1 to Day +7 of vaccination among the study participants were low. Many participants dropped out or skipped one or a few days of the surveys. In addition, we could not evaluate the potential impact of non‐responders. This has potentials of both underestimating and overestimating the true impacts because both those with very severe adverse events (i.e., too severe symptoms to answer the survey) and those without any recognizable adverse events (i.e., those with no symptoms may have forgotten to answer the survey) could be non‐responders or drop outs. Second, the sample size of the subgroup analysis was small. Some data in the subgroup analysis (e.g., −0.016 mean QALY loss in those with grade 1 AEFI) should be interpreted with caution. Third, this study used the pre‐vaccination status of vaccinees as reference to calculate QALY loss same as previous studies which evaluated QALY changes following vaccination using the EQ‐5D instrument [17, 19]. With the lack of inclusion of the unvaccinated population as a control group, we could not evaluate the potential impact of vaccine‐unrelated daily fluctuations on the QALY change of vaccinees.

In conclusion, we measured the magnitude of QALD loss in HCWs following inactivated influenza vaccination. Although reactogenicity symptoms resolved within a few days following vaccination, severe‐grade AEFI was associated with a larger QALD loss. These results support a more accurate health technology assessment of seasonal influenza vaccination in this population.

## Author Contributions

**Taito Kitano:** conceptualization, methodology, software, data curation, investigation, validation, formal analysis, visualization, project administration, resources, writing – original draft. **Sayaka Yoshida:** conceptualization, methodology, supervision, visualization, project administration, resources, writing – review and editing.

## Ethics Statement

The study was approved by the Institutional Review Board of Nara Prefecture General Medical Center (Approval No. 923‐2).

## Consent

A consent statement was included in the introduction to the survey. Participants provided consent by answering the survey with reading the consent statement. Participants were informed that participation in the survey was voluntary and that the survey results would be published.

## Conflicts of Interest

The authors declare no conflicts of interest.

## Data Availability

The data supporting the findings of this study are available upon reasonable request.
